# Effects of 5-aminolevulinic acid and sodium ferrous citrate on fibroblasts from individuals with mitochondrial diseases

**DOI:** 10.1038/s41598-019-46772-x

**Published:** 2019-07-22

**Authors:** Masaru Shimura, Naoko Nozawa, Minako Ogawa-Tominaga, Takuya Fushimi, Makiko Tajika, Keiko Ichimoto, Ayako Matsunaga, Tomoko Tsuruoka, Yoshihito Kishita, Takuya Ishii, Kiwamu Takahashi, Tohru Tanaka, Motowo Nakajima, Yasushi Okazaki, Akira Ohtake, Kei Murayama

**Affiliations:** 1 Center for Medical Genetics, Department of Metabolism, Chiba Children’s Hospital, 579-1 Heta-cho, Midori-ku, Chiba, 266-0007 Japan; 2 Division of Pharmaceutical Research, SBI Pharmaceuticals Co., Ltd., 1-6-1 Roppongi, Minato-ku, Tokyo, 106-6020 Japan; 3 Intractable Disease Research Center, Graduate School of Medicine, Juntendo University, 2-1-1, Hongo, Bunkyo-ku, Tokyo, 113-8421 Japan; 4 Department of Pediatrics & Clinical Genomics, Faculty of Medicine, Saitama Medical University, 38 Morohongo, Moroyama, Saitama 350-0495 Japan; 5 Center for Intractable Diseases, Saitama Medical University Hospital, 38 Morohongo, Moroyama, Saitama 350-0495 Japan

**Keywords:** Energy metabolism, Neurological disorders, Drug development

## Abstract

Mitochondrial respiratory chain complexes II, III, and IV and cytochrome c contain haem, which is generated by the insertion of Fe^2+^ into protoporphyrin IX. 5-Aminolevulinic acid (ALA) combined with sodium ferrous citrate (SFC) was reported to enhance haem production, leading to respiratory complex and haem oxygenase-1 (HO-1) upregulation. Here, we investigated the effects of different concentrations of ALA and SFC alone or in combination (ALA/SFC) on fibroblasts from 8 individuals with mitochondrial diseases and healthy controls. In normal fibroblasts, expression levels of oxidative phosphorylation (OXPHOS) complex subunits and corresponding genes were upregulated only by ALA/SFC. Additionally, the increased oxygen consumption rate (OCR) and ATP levels in normal fibroblasts were more obvious after treatment with ALA/SFC than after treatment with ALA or SFC. OXPHOS complex proteins were enhanced by ALA/SFC, whereas OCR and ATP levels were increased in 6 of the 8 patient-derived fibroblasts. Further, HO-1 protein and mRNA levels were enhanced by ALA/SFC in all fibroblasts. The relative mtDNA copy number was increased by ALA/SFC. Thus, our findings indicate that ALA/SFC is effective in elevating OXPHOS, HO-1 protein, and mtDNA copy number, resulting in an increase in OCR and ATP levels, which represents a promising therapeutic option for mitochondrial diseases.

## Introduction

Mitochondrial diseases are clinically and genetically heterogeneous disorders caused by defects of energy production via oxidative phosphorylation (OXPHOS). More than 1500 genes, encoded by nuclear and mitochondrial DNA, are involved in mitochondrial function. The perspective on mitochondrial diseases is being clarified with identification of multiple causative genes with new genetic technologies. Although various treatments for mitochondrial diseases have been suggested, none has been supported with sufficient evidence^1^.

**Figure 1:**
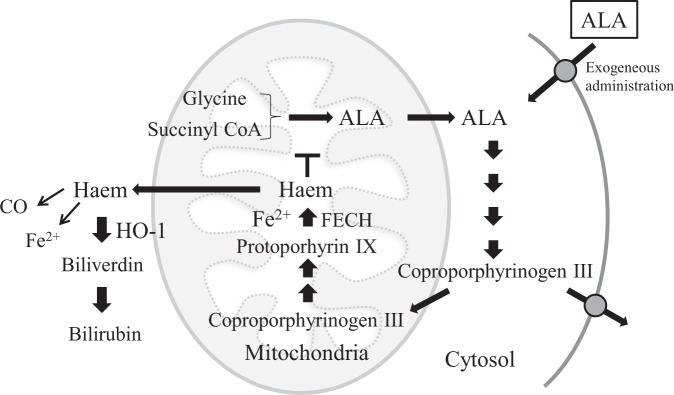
Haem biosynthesis pathway. Haem is biosynthesised via several steps in the cytoplasm and mitochondria. 5-aminolevulinic acid (ALA) as an endogenous amino acid is generated from glycine and succinyl CoA. Through the porphyrin pathway, ALA is converted to protoporphyrin IX. Haem is generated by the insertion of ferrous ion into protoporphyrin IX. Exogenously administered ALA is absorbed into the cytoplasm, and then used as a substrate for protoporphyrin IX. Haem is degraded to carbon monoxide (CO), iron, and biliverdin by haem oxygenase-1 (HO-1).

Haem, which is synthesised by the insertion of ferrous ion into protoporphyrin IX, acts as a protein-bound prosthetic group in mitochondrial respiratory chain complexes II, III, and IV, and cytochrome C (Fig. 1). 5-Aminolevulinic acid (ALA) is an endogenous amino acid and an intermediate in haem biosynthesis, generated by condensation of glycine and succinyl CoA. Since ALA causes tumour accumulation of protoporphyrin IX, it has already been used as an oral diagnostic drug for cancer in Japan, Europe, Australia, and Canada^2,3^. We previously reported that ALA supplementation in mice enhances cytochrome *c* oxidase (COX) activity and ATP production levels in liver, and ALA combined with sodium ferrous citrate (SFC) administration upregulates the expression of OXPHOS complexes III, IV, and V in white adipose tissues of diet-induced obese mice, resulting in reduced lipid content of adipocytes^4,5^. Furthermore, ALA combined with SFC in healthy humans increased the expression levels of haem oxygenase-1 (HO-1) in peripheral blood mononuclear cells, which has cytoprotective effects against oxidative stress and inflammation^6,7^.

Taken together, it is assumed that ALA combined with SFC (ALA/SFC) treatment for patients with mitochondrial diseases improves mitochondrial respiratory chain functions, leading to an increase in ATP production, which ameliorates disease symptoms. In the present study, we investigated the effects of ALA, SFC, or combined ALA/SFC administration on skin fibroblasts from individuals with mitochondrial diseases.

## Results

### OXPHOS protein and gene expression levels in normal human skin fibroblasts

**Figure 2:**
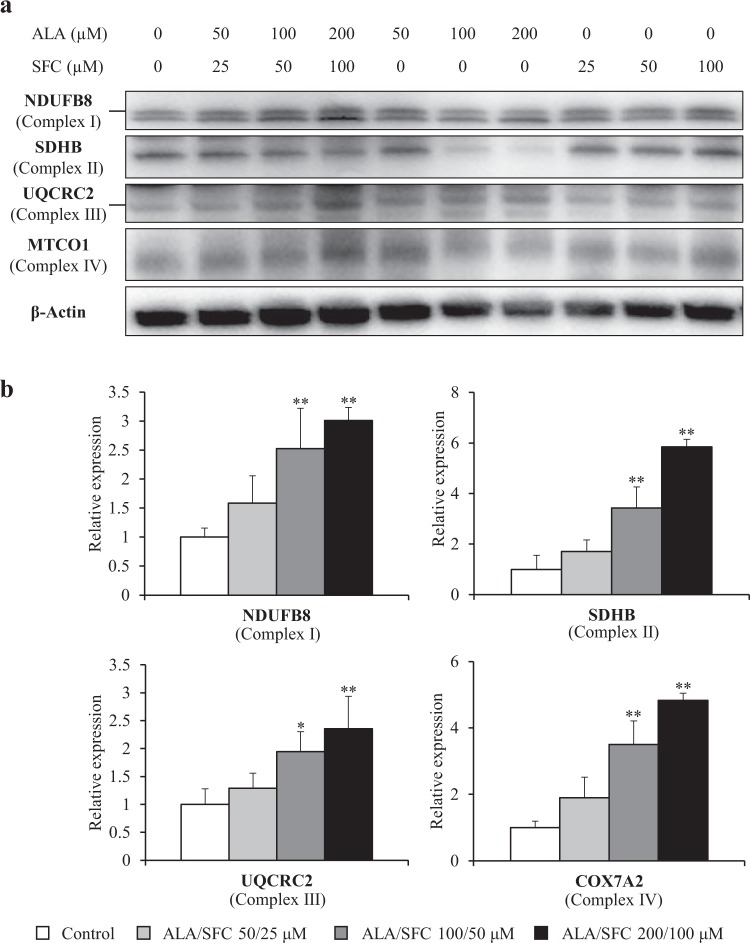
Protein levels and gene expressions of OXPHOS complex subunits in normal human skin fibroblasts. (**a**) All OXPHOS complex proteins were upregulated by ALA/SFC. ALA or SFC alone did not enhance protein expression. Cropped blots are displayed. Full-length blots are presented in Supplementary Fig. 1. (**b**) ALA/SFC significantly increased gene expression of OXPHOS complex subunits in a concentration-dependent manner. Data are expressed as means ± SD of 3 independent experiments, relative to the control, * *P* < 0.05, **P < 0.01 vs. Control (Dunnett’s test).

We first evaluated the effects of ALA, SFC, and ALA/SFC on OXPHOS protein expression in normal human skin fibroblasts. The protein levels of NDUFB8 (complex I), UQCRC2 (complex III), and MTCO1 (complex IV) were increased in the normal control cells cultured with ALA/SFC in a concentration-dependent manner. In contrast, cells cultured with ALA and with SFC alone did not show significant elevations in these protein levels (Fig. 2a). The corresponding gene expression levels in control fibroblasts treated with ALA/SFC were determined by quantitative reverse transcription PCR. Consistent with the western blot analysis, the gene expression levels of OXPHOS were enhanced by ALA/SFC (*P* < 0.05) (Fig. 2b). The relative gene expression levels with ALA/SFC 200/100 µM were ~2 to 6 times higher than those in the control cells without administration.

### Oxygen consumption rate (OCR) and ATP production levels in normal human skin fibroblasts

**Figure 3:**
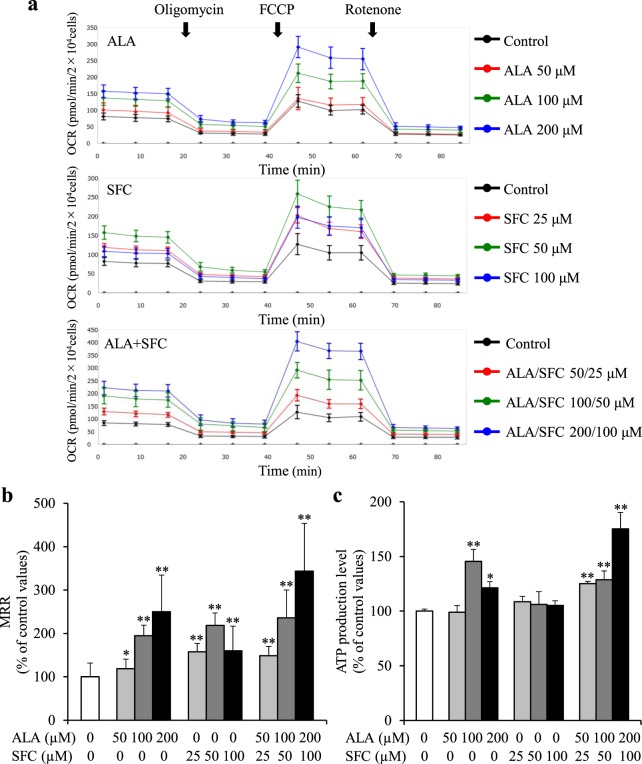
Oxygen consumption rate profile and ATP production levels of normal human skin fibroblasts. (**a**) Oxygen consumption rate (OCR) was enhanced by not only ALA/SFC but also by ALA or SFC in normal fibroblasts. ALA/SFC is the most effective at increasing OCR. Data are expressed as means ± SD of > 22 technical replicates. (**b**) Maximum respiration rates (MRRs) were increased by all treatments. Data are expressed as means ± SD of >22 technical replicates, relative to the control. *P < 0.05, **P < 0.01 vs. Control (Dunnett’s test). (**c**) ATP levels were significantly increased by ALA and ALA/SFC. Data are expressed as means ± SD of 3 independent experiments, relative to the control, *P < 0.05, **P < 0.01 vs. Control (Dunnett’s test).

We performed oxygen consumption analysis using normal human skin fibroblasts, treated with ALA-, SFC-, and ALA/SFC-containing medium, to investigate the impact of each treatment. The OCR was remarkably increased by all treatments at all doses tested (Fig. 3a). The results of maximum respiration rate (MRR) in comparison with each control value are shown in Fig. 3b. The MRR in fibroblasts treated with ALA or ALA/SFC increased in a concentration-dependent manner. Similarly, the reserve capacity and proton leak levels showed concentration-dependent increases with ALA or ALA/SFC treatment (Fig. S2). Interestingly, cells treated with 50 µM SFC showed significant increases compared with those treated with 100 µM SFC. ALA/SFC 200/100 µM administration was the most effective in improving OCR, showing a 3.4-fold increase in MRR compared with that of the control (*P* < 0.01). Consistent with the OCR findings, ATP production levels were significantly increased 1.7-fold by ALA/SFC 200/100 µM (*P* < 0.01) (Fig. 3c). SFC alone did not produce an increase in ATP production. As is evident from the above results, ALA/SFC was considered the most effective treatment for fibroblasts from individuals with mitochondrial diseases.

### OXPHOS protein expression in patient-derived fibroblasts

**Figure 4:**
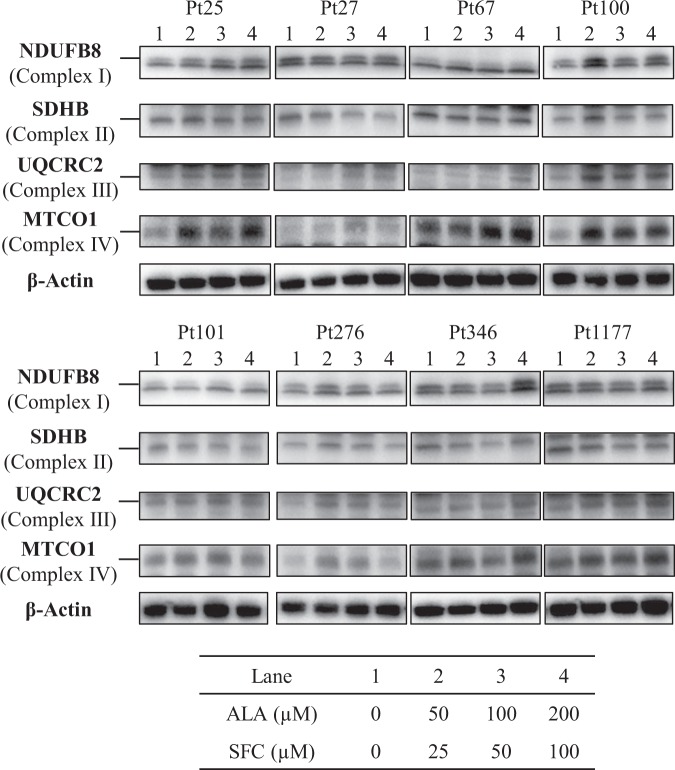
Western blot analysis of OXPHOS complex subunits in fibroblasts from patients with mitochondrial diseases. UQCRC2 (complex III) and MTCO1 (complex IV) levels were enhanced by ALA/SFC in fibroblasts from all patients. NDUFB8 (complex I) levels were upregulated in 5/8 fibroblasts from Pt25, Pt100, Pt101, Pt276, and Pt346, and SDHB (complex II) levels were increased in Pt100 and Pt276. Cropped blots are displayed. Full-length blots are presented in Supplementary Figs 3–5.

To evaluate the impact of ALA/SFC on OXPHOS protein expression in patient-derived fibroblasts, we conducted western blot analyses of OXPHOS complex subunits. Complex III and complex IV subunits were increased in fibroblasts from all patients, while complex I subunit levels were increased in 5/8 fibroblasts from Pt25, Pt100, Pt101, Pt276, and Pt346 (Fig. 4). In contrast, increases in complex II subunit levels were observed only in Pt100 and Pt276. Some fibroblasts showed an increase in OXPHOS protein expression that was proportional to the treatment concentration, but others showed an increase that was concentration-independent.

### OCR and ATP production levels in patient-derived fibroblasts

**Figure 5:**
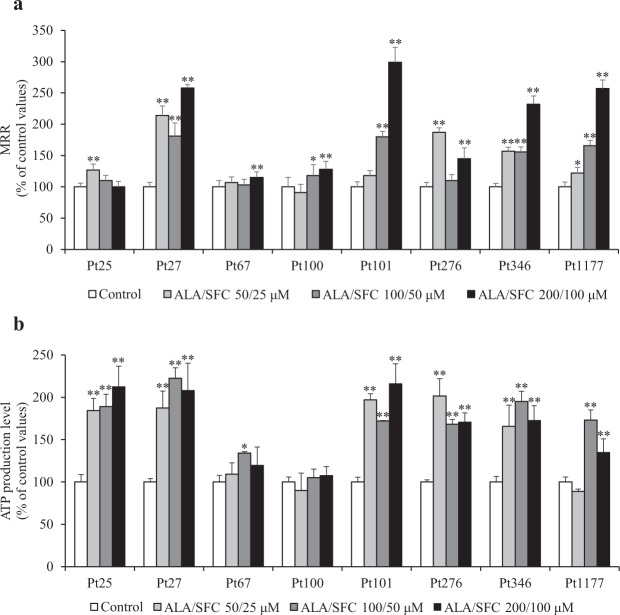
Maximum respiration rates and ATP production levels in fibroblasts from patients with mitochondrial diseases. (**a**) Maximum respiration rates were significantly increased by ALA/SFC in fibroblasts from all patients. Data are expressed as means ± SD of >14 technical replicates, relative to the control. *P < 0.05, **P < 0.01 vs. Control (Dunnett’s test). (**b**) ALA/SFC significantly enhanced ATP production in fibroblasts from 7 of 8 patients. Data are expressed as means ± SD of 3 technical replicates, relative to the control, *P < 0.05, **P < 0.01 vs. Control (Dunnett’s test).

We performed oxygen consumption analysis on fibroblasts treated with ALA/SFC from individuals with mitochondrial diseases. MRR levels were determined and compared with the levels from corresponding fibroblasts without treatment (Fig. 5a). MRR levels in all patient-derived fibroblasts were upregulated by ALA/SFC (*P* < 0.05). Half of them showed a 2–3-fold upregulation of MRR relative to controls. The reserve capacity and proton leak were increased in 6 of 8 patient-derived fibroblasts treated with ALA/SFC (Fig. S6). The results of ATP production levels are shown in Fig. 5b. Seven out of eight fibroblasts showed a 1.7–2.1-fold increase in ATP levels with ALA/SFC treatment (*P* < 0.05).

### HO-1 protein levels and gene expression

**Figure 6:**
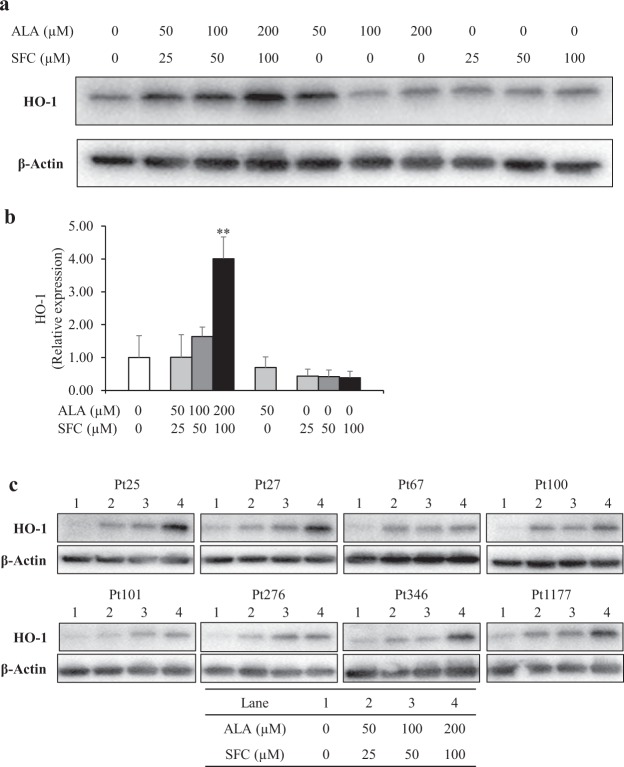
HO-1 protein levels and gene expression in skin fibroblasts. (**a**) ALA/SFC remarkably increased HO-1 expression in normal skin fibroblasts, whereas ALA or SFC alone did not upregulate its protein expression. Cropped blots are displayed. Full-length blots are presented in Supplementary Fig. 7. (**b**) In normal human skin fibroblasts, ALA/SFC increased *HO-1* gene expression in a concentration-dependent manner. The expression levels with ALA 100 µM and 200 μM were not determined due to insufficient numbers of viable cells. Data are expressed as means ± SD of 3 independent experiments, relative to the control, **P < 0.01 vs. Control (Dunnett’s test). (**c**) ALA/SFC enhanced HO-1 protein expression in fibroblasts from all patients. Cropped blots are displayed. Full-length blots are presented in Supplementary Fig. 8.

We previously reported that ALA/SFC enhances HO-1 expression, which has anti-inflammatory and antioxidant activities, in a mouse fatty liver model, and in peripheral blood mononuclear cells of healthy humans^6,8^. We therefore carried out western blot analysis and quantitative reverse transcription PCR of the *HO-1* gene. We first investigated the effect of ALA, SFC, or combined ALA/SFC on normal human skin fibroblasts. Western blot analysis showed that HO-1 protein levels were elevated by ALA/SFC in a concentration-dependent manner, but not by ALA or SFC alone (Fig. 6a). Additionally, *HO-1* mRNA levels were remarkably upregulated by ALA/SFC in a concentration-dependent manner (*P* < 0.01) (Fig. 6b). Consistent with the findings in normal human skin fibroblasts, HO-1 protein levels in fibroblasts from all individuals with mitochondrial diseases were also concentration-dependently increased by ALA/SFC treatment (Fig. 6c).

### Analysis of mtDNA copy number

**Figure 7:**
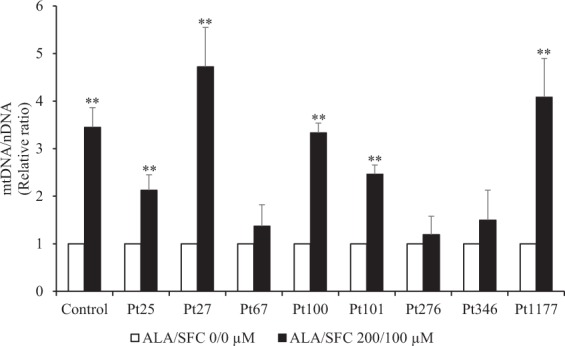
Relative mtDNA copy number in skin fibroblasts. The mtDNA/nDNA ratio was significantly increased by ALA/SFC in control fibroblasts and 5 of 8 patient-derived fibroblasts. Data are expressed as means ± SD of 3 independent experiments, relative to each fibroblast without ALA/SFC treatment, **P < 0.05 (Student’s t test).

HO-1 has been shown to promote mitochondrial biogenesis^9^. We performed quantitative PCR to assess the change in mitochondrial DNA (mtDNA) copy number relative to nuclear DNA (nDNA) in skin fibroblasts with or without ALA/SFC treatment (ALA/SFC 200/100 µM). The mtDNA/nDNA ratio was significantly increased in normal human skin fibroblasts and 5 of 8 patient-derived fibroblasts treated with ALA/SFC (Fig. 7).

## Discussion

To the best of our knowledge, this is the first study to investigate the effects of ALA and SFC on skin fibroblasts from individuals with mitochondrial diseases. We have shown that ALA/SFC enhances OXPHOS complex protein levels and gene expression in normal human skin fibroblasts as well as those in patient-derived fibroblasts. In addition, our study demonstrated that ALA/SFC increases OCR and ATP production levels, and upregulates HO-1 protein levels and gene expression in both fibroblast types.

We first observed that ALA/SFC successfully upregulated OXPHOS proteins with increased OXPHOS gene expression, whereas ALA or SFC alone could not upregulate these proteins. These findings are consistent with several previous studies showing that a combination of ALA and SFC was necessary to produce haem proteins^5,10–12^. Respiratory chain complexes II, III, and IV and cytochrome C contain haem; thus, an upregulation of haem by ALA/SFC might contribute to OXPHOS protein expression. Regarding mitochondrial oxygen consumption, ALA or SFC alone as well as ALA/SFC increased OCR. Possible explanations for this finding include the following. Our previous study showed that ALA increases haem production, resulting in COX activation and an increase in ATP levels in mouse liver^4^. Iron deficiency or excess has been reported to cause damage to mitochondria and mitochondrial DNA^9^. However, it has been demonstrated that iron supplementation increases the activities of four enzymes of the Krebs cycle, namely aconitase, citrate synthase, isocitrate dehydrogenase, and succinate dehydrogenase, while iron chelation reduces their activities^13^. Hence, SFC might result in increased formation of nicotinamide adenine dinucleotide via the Krebs cycle, leading to increased mitochondrial oxygen consumption without upregulation of OXPHOS proteins.

In patient fibroblasts, although there is variability among patients, all OXPHOS complex proteins were enhanced by ALA/SFC. Of note, although complex I does not contain haem, complex I (NDUFB8) protein expression was enhanced. NDUFB8 does not have an iron-sulphur cluster and therefore its expression may not be affected by iron. One plausible reason for the increase in NDUFB8 expression is the increased expression of HO-1. HO-1 is a stress-responsive gene induced by oxidative stress or other stimuli, and it has various functions. Several studies have implicated HO-1 in the regulation of mitochondrial quality control, fission and fusion, biogenesis, and mitophagy^12,14,15^. Thus, it is possible that an upregulation of HO-1 exerts an effect on the expression of complex I (NDUFB8).

OCR and ATP levels were remarkably enhanced by ALA/SFC in most fibroblasts, whereas fibroblasts from Pt67 and Pt100 showed limited or no significant effects. Pt67 had a missense mutation in *NDUFB11* encoding a complex I component. Whole exome sequencing identified a heterozygous variant, c.580G > A, in *NDUFV2* that was identified as the causal variant in Pt1177 in our previous report^16^. Although Pt100 had no other rare exonic variant in *NDUFV2*, RNA sequencing of Pt100 fibroblast cells revealed that mRNA expression of the c.580G wild-type allele was apparently decreased (22–30% of c.580A). Therefore, we believe that *NDUFV2* is a strong candidate as a causal gene. There was no NDUFB11 protein expression in Pt67 fibroblasts^17^. Moreover, Pt67 had a severe clinical course, including heart failure and respiratory failure, resulting in death 55 h after birth^17^. Other individuals survived at least 4 months. Collectively, the limited increases in OCR and ATP levels might be associated with the remarkable reduction in encoded proteins and with the severe phenotype.

Haem metabolism plays a central role in diverse functions of the mitochondria^9^. Impaired haem homeostasis is reportedly associated with pathophysiological conditions in several neurodegenerative disorders; for example, excess free haem resulting from intracerebral or subarachnoid haemorrhages promotes oxidative stress, lipid peroxidation, inflammatory response, and neuronal cell death^9^. In contrast, haem deficiency results in defective neurite outgrowth and cell apoptosis. Therefore, the regulation of intracellular and extracellular haem homeostasis is strictly maintained by several mechanisms^18^. HO-1 is induced by a variety of stimuli such as oxidative stress, hypoxia, ischaemia-reperfusion, cytokines, and its substrate, haem^19^. HO-1 is a haem-catabolizing enzyme, which degrades intracellular haem to produce iron, carbon monoxide (CO), and biliverdin, which is converted into bilirubin by biliverdin reductase (Fig. 1)^6,18^. HO-1, HO-1-derived bilirubin, biliverdin, and CO have been shown to have antioxidative and anti-inflammatory properties^11,20^. Furthermore, HO-1 and CO, which can be increased by ALA/SFC, have been shown to increase mtDNA copy number by enhanced protein expression of nuclear respiratory factor-1 (NRF1), peroxisome proliferator-activated receptor-γ coactivator 1-α (PGC1α), and mitochondrial transcriptional factor A (TFAM)^8,14,21,22^. In this study, we observed that ALA/SFC upregulated mtDNA copy number, as well as *HO-1* mRNA and protein in normal and patient-derived fibroblasts. These findings indicate that ALA/SFC may exert a protective effect on fibroblasts and promote mitochondrial biogenesis via an increase in HO-1 and CO in patients with mitochondrial diseases.

In recent years, haem metabolism has been attracting attention as a promising strategy for the treatment of neurodegenerative diseases. Particularly, HO-1 has received considerable attention due to its well-established neuroprotective role^9^. The protective role of HO-1 upregulation has been confirmed in animal models of Parkinson’s disease, Alzheimer’s disease, brain ischaemia, and traumatic brain injury^23,24^. However, the effects of ALA/SFC-induced upregulation of haem and HO-1 on mitochondrial diseases have never been investigated. Therefore, this study is helpful for understanding their mechanism and impact on mitochondrial diseases. Although various clinical trials of treatments for mitochondrial diseases have been carried out, there are few treatments available with sufficiently strong evidence of efficacy. A clinical trial of ALA/SFC treatment for Leigh syndrome (LS) was conducted in Japan. We have observed some improvements in clinical symptoms and it is expected that ALA/SFC treatment will be put into practical use soon.

Several limitations of this study should be mentioned. First, we could not evaluate reactive oxygen species (ROS) levels associated with the upregulated ATP production levels. ROS are involved in oxidative damage in a range of pathologies and redox signalling^25^; thus, it is possible that an alteration of ROS by ALA/SFC had an impact on the results. Second, this study was conducted using only skin fibroblasts. It has been reported that overexpression of HO-1 induces oxidative mitochondrial damage and macroautophagy in cultured rat astroglia^9,26,27^. Additional work is required to reveal the impact of ALA/SFC on other tissues. Third, the number of samples was small. Mitochondria contain approximately 1500 different proteins, while only approximately 300 causative genes have been reported to date. Additionally, the effect of ALA/SFC can differ among variants, even of the same gene. Further studies are required to elucidate the effects of ALA/SFC with different causative genes and variants.

In summary, our findings suggest that treatment with ALA/SFC increases OCR and ATP levels via OXPHOS protein expression induced by an upregulation of haem, HO-1, and mtDNA copy number in skin fibroblasts from individuals with mitochondrial diseases. These *in vitro* results provide strong evidence for the practical use of ALA/SFC treatment for mitochondrial diseases.

## Methods

This study protocol using skin fibroblasts from individuals with mitochondrial diseases was approved by the ethics boards of Chiba Children’s Hospital and Saitama Medical University. All experiments were performed in accordance with relevant guidelines and regulations. Written informed consent was obtained from parents of all subjects.

### Subjects

The study was performed on skin fibroblasts from 8 Japanese individuals (5 males and 3 females) diagnosed with mitochondrial diseases. The characteristics of the subjects are shown in Table 1. Pt25, Pt67, Pt101, Pt276, and Pt346 were previously reported^17,28^. All individuals were enzymatically diagnosed. We included 6 subjects with complex I deficiency in the 8 subjects because complex I deficiency is the most common enzyme defect in Japan^1^, and we aimed to enhance ATP production through upregulation of complexes II, III, and IV, as well as cytochrome C via ALA/SFC treatment, thus bypassing complex I. Causative genes were identified in 7 out of the 8 cases. Their clinical diagnoses included 3 individuals with LS, 3 with infantile mitochondrial diseases (IMD), 1 with neurodegenerative disorder (ND), and 1 with mitochondrial hepatopathy (MH).

**Table 1:** Patient characteristics.

No.	ID	Sex	Age of onset	Clinical diagnosis	Affected complex	Gene	Mutations
1	Pt25	F	5 m	IMD	CI	*ACAD9*	c.811T > G:p.C271G/ c.1766-2A > G
2	Pt27	M	1 y	LS	CIV	*SURF1*	c.743C > A:p.A248D/ c.743C > A:p.A248D
3	Pt67	M	0 d	IMD	CI	*NDUFB11*	c.391G > A:p.E131K (hemizygous)
4	Pt100	M	8 m	ND	CI	*NDUFV2*	c.580G > A:p.E194K/ unknown
5	Pt101	M	11 m	LS	CI	*NDUFAF6*	c.371T > C:p.I124T/ c.805C > G:p.H269D
6	Pt276	M	1 y 11 m	MH	CI + IV	*MRPS23*	c.119C > G:p.P40R/ c.119C > G:p.P40R
7	Pt346	F	0 d	IMD	CI	*ECHS1*	c.176A > G:p.N59S/ c.476A > G:p.Q159R
8	Pt1177	F	9 m	LS	CI	*NDUFV2*	c.427C > T:p.R143X/ c.580G > A:p.E194K

C; complex, IMD; infantile mitochondrial diseases, LS; Leigh syndrome, ND; neurodegenerative disorder, MH; mitochondrial hepatopathy.

### Cell culture

Skin fibroblasts from eight individuals and control fibroblasts (NHDF-neo; Lonza) were cultured at 37 °C (5% CO_2_) in Dulbecco’s modified Eagle’s medium (DMEM; Lonza) supplemented with 10% foetal bovine serum (FBS) and 1% penicillin/streptomycin. Control fibroblasts at passage 7 and patient-derived fibroblasts at passages 4 to 10 were used for all experiments. The fibroblasts were seeded in flasks and 96-well plates for ATP determination at 2 × 10^4^ cells/well with 80 µL of growth medium containing 25 mM glucose and incubated for 24 h (37 °C, 5% CO_2_). The medium was then replaced with culture medium supplemented with 10% FBS and four different concentrations of ALA/SFC: 0/0 μM, 50/25 μM, 100/50 μM, or 200/100 μM (ALA: neo ALA corporation, Tokyo, Japan; SFC: Komatsuya Corporation, Osaka, Japan). In addition, control fibroblasts were cultured with ALA- (50, 100, 200 µM), SFC- (25, 50, 100 µM), or ALA/SFC- (50/25, 100/50, 200/100 µM) containing medium to evaluate the effect of each treatment. All experiments were conducted under dark conditions owing to the instability of SFC in light. The culture media containing ALA, SFC, or ALA/SFC were replaced every 2 days for a period of 10 days.

### Western blotting

Western blotting was performed by a modification of Hara’s method and Ota’s method^5,29^. In particular, total protein extracts (2 × 10^4^ cells/lane) were separated by polyacrylamide gel electrophoresis, transferred to polyvinylidene difluoride membranes using the Trans-Blot^®^ Turbo Transfer system (Bio Rad Laboratories, USA), and incubated with antibodies. Immuno-labelled proteins were detected using a chemiluminescence kit (Immuno Star LD (FUJIFILM Wako Pure Chemical, Japan)) and a lumino-image analyser (ChemiDoc MP system (Bio Rad Laboratories, USA)). The primary antibodies used were Total OXPHOS Rodent WB Antibody Cocktail (abcam, UK) and rabbit anti-human HO-1 antibody (kindly provided by Dr. Shigeru Taketani)^30,31^. The secondary antibodies used were anti-rabbit IgG HRP-Linked Whole Ab Donkey (GE Healthcare, USA) for OXPHOS proteins and anti-mouse IgG HRP-Linked Whole Ab Sheep (GE Healthcare, USA) for HO-1. Beta-actin was used as an internal control. Anti-beta-actin antibody (ab8227) (abcam, UK) was used as primary antibody and Anti-Rabbit IgG HRP-Linked Whole Ab Donkey (GE Healthcare, USA) was used as secondary antibody.

### Quantitative reverse transcription-PCR analysis

Total RNA of the cells treated with ALA/SFC was purified according to the protocol provided with the RNeasy Mini kit (50) (QIAGEN, Germany). The amount and purity of the total RNA were measured using the NanoDrop One spectrophotometer (Thermo Scientific, USA). cDNA was synthesised from 1 μg of total RNA using the High Capacity RNA-to-cDNA kit (Life Technologies, USA). The expression level of each gene was measured using the SYBR^®^ Select Master Mix (Life Technologies, USA) and the StepOnePlus Real-Time PCR System (Life Technologies, USA). The sequences of the primers used are described in Supplementary Table 1. Beta-2-microglobulin (B2M) was used as an internal control.

### Real-time PCR for quantitative measurement of mtDNA

Quantitative PCR was performed to evaluate the change in mtDNA copy number in skin fibroblasts treated with or without ALA/SFC (ALA/SFC 200/100 µM), as previously described^32,33^. The real-time amplification of the mtDNA gene *MT-ND1* was compared to that of a single copy nuclear reference gene (exon 24 of the *CFTR* gene). The results presented are the means of three independent runs, with samples assayed in triplicate in each run.

### Measurement of OCR

OCR was measured with the XF96 extracellular flux analyser (Seahorse XF96 system; Seahorse Bioscience, Billerica, MA, USA). Materials were prepared using previously reported methods^34^. Skin fibroblasts cultured with ALA, SFC, or combined ALA/SFC were seeded in a 96-well plate at 2 × 10^4^ cells/well with 80 µL of growth medium containing 25 mM glucose, and incubated for 24 h (37 °C, 5% CO_2_). After replacing the medium with 160 µL of un-buffered DMEM containing 25 mM glucose, 1 mM sodium pyruvate, and 2 mM glutamine, the assay plates were incubated at 37 °C without CO_2_ for 1 h. Following the calibration of the sensor cartridge loaded with compounds including oligomycin (2 µM final), carbonyl cyanide 4-(trifluoromethoxy) phenylhydrazone (FCCP, 0.4 µM final), and rotenone (1 µM final), the experiments were started. The data obtained were normalised to the cell numbers determined using the CyQUANT^®^ Cell Proliferation kit (Invitrogen). The MRR corresponds to the OCR after the addition of FCCP minus rotenone-insensitive OCR. The reserve capacity (spare respiratory capacity), which indicates the ability of the cell to respond to ATP demand, was calculated as maximal respiration minus basal respiration^35^. The proton leak, which increases with membrane potential, was calculated as the difference between the OCR after FCCP injection and rotenone-insensitive OCR^36^. Each parameter was evaluated in comparison with the corresponding fibroblasts without treatment.

### Measurement of ATP level

Intracellular ATP levels were determined using the ATPlite Assay kit (Perkin Elmer) according to the slightly modified standard procedure. The method is based on the luciferin-luciferase reaction with ATP. Following ALA/SFC treatment, the medium in the 96-well plates was removed and the cells were washed with 200 µL of PBS. After adding 25 µL of mammalian cell lysis solution and 50 µL of PBS, the plates were shaken for 5 min at 700 rpm. Then, 37.5 µL of sample and 12.5 µL of substrate solution were added to the white-wall, clear-bottom 96-well plate, shaken for 5 min at 700 rpm, and incubated for 10 min in the dark. The luminescence levels were measured using a plate reader (Tecan Japan). ATP levels were corrected by the number of cells determined using the CyQUANT^®^ Cell Proliferation kit (Invitrogen) and evaluated in comparison with the corresponding fibroblasts without treatment.

### Statistical analysis

Data obtained are expressed as the means of the replicates ± standard deviation (SD). Statistical analysis was performed using Dunnett’s test, and *P* values < 0.05 were considered statistically significant.

## Supplementary information

Supplementary Information
