# Supplementary material for: Effects of 5-aminolevulinic acid and sodium ferrous citrate on fibroblasts from individuals with mitochondrial diseases
Source: Sci Rep. 2019 Jul 22;9:10549. doi: 10.1038/s41598-019-46772-x (PMC6646320; doi:10.1038/s41598-019-46772-x)
Supplement: Supplementary file 1 — Supplementary Information [file 41598_2019_46772_MOESM1_ESM.docx]

**Supplementary Information**

**Effects of 5-aminolevulinic acid and sodium ferrous citrate on fibroblasts from individuals with mitochondrial diseases**

Masaru Shimura^1^, Naoko Nozawa^2^, Minako Ogawa-Tominaga^1^, Takuya Fushimi^1^, Makiko Tajika^1^, Keiko Ichimoto^1^, Ayako Matsunaga^1^, Tomoko Tsuruoka^1^, Yoshihito Kishita^3^, Takuya Ishii^2^, Kiwamu Takahashi^2^, Tohru Tanaka^2^, Motowo Nakajima^2^, Yasushi Okazaki^3^, Akira Ohtake^4^, Kei Murayama^1*^

^1^Center for Medical Genetics, Department of Metabolism, Chiba Children’s Hospital, 579-1 Heta-cho, Midori-ku, Chiba 266-0007, Japan

^2^Division of Pharmaceutical Research, SBI Pharmaceuticals Co., Ltd., 1-6-1 Roppongi, Minato-ku, Tokyo 106-6020, Japan

^3^Intractable Disease Research Center, Graduate School of Medicine, Juntendo University, 2-1-1, Hongo, Bunkyo-ku, Tokyo 113-8421 Japan

^4^Department of Pediatrics, Faculty of Medicine, Saitama Medical University, 38 Morohongo, Moroyama, Saitama 350-0495, Japan

***Corresponding author:**

Dr. Kei Murayama

Center for Medical Genetics, Department of Metabolism, Chiba Children’s Hospital, 579-1 Heta-cho, Midori-ku, Chiba 266-0007, Japan

Email: [kmuraya@mri.biglobe.ne.jp](mailto:kmuraya@mri.biglobe.ne.jp); Tel: +81-43-292-2111; Fax: +81-43-292-3815

**Supplementary Table 1.** Primer Sequences (5′–3′)

| Target Gene | Forward | Reverse |
| --- | --- | --- |
| *NDUFB8* | CATGGGGTATGGCGACTACC | CGGTTCACCCCAGTTCAACC |
| *SDHB* | GCTACTGGTGGAACGGAGAC | GGTGTGGCAGCGGTATAGAG |
| *UQCRC2* | CAACATGAGGGGTGGGCTTG | CTTCCCGCGACAGCACTTTC |
| *COX7A2* | AGATTGGGCAGAGGACGATAA | CCCACCCTTTAGATACAGTGGAA |
| *ATP5A1* | GGAGCTGTTGGGTCGTGTAG | GATACCGGGGGCTTTCAGAC |
| *HO-1* | ACGGCTTCAAGCTGGTGATG | CGAAGACTGGGCTCTCCTTG |
| *B2M* | TGCTGTCTCCATGTTTGATGTATCT | TCTCTGCTCCCCACCTCTAAGT |


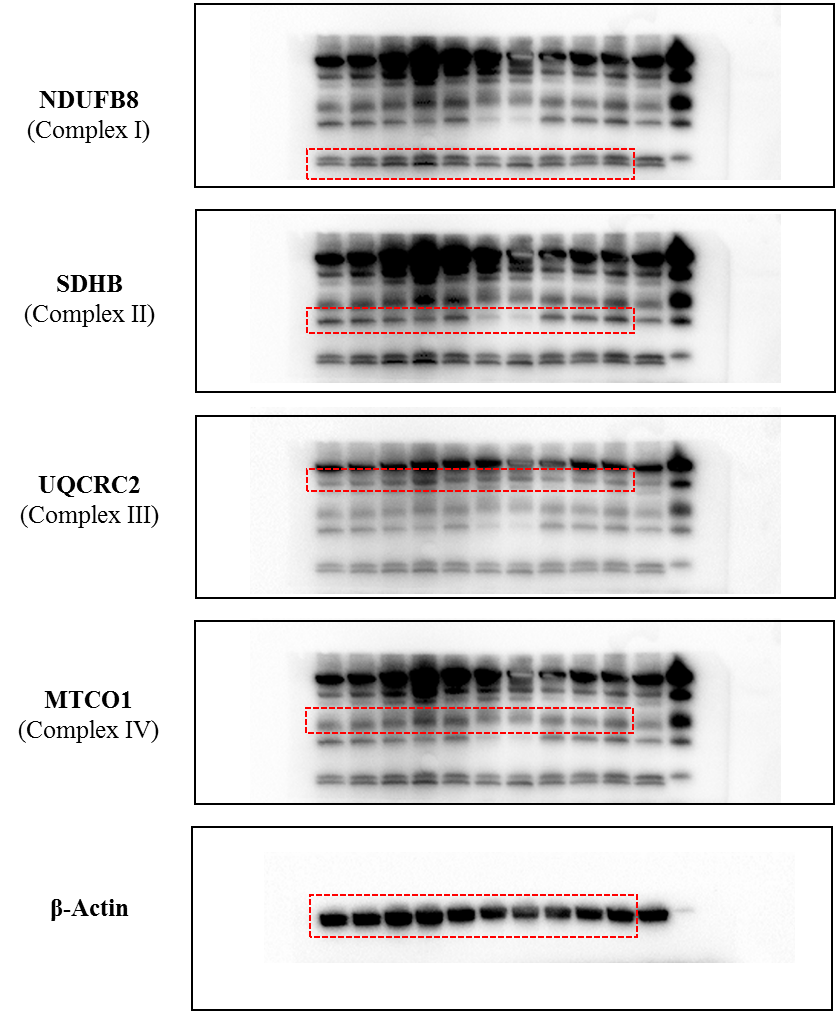


**Supplementary Figure 1. Full-length blots of Figure 2a**

Red boxes indicate cropped regions shown in Figure 2a.


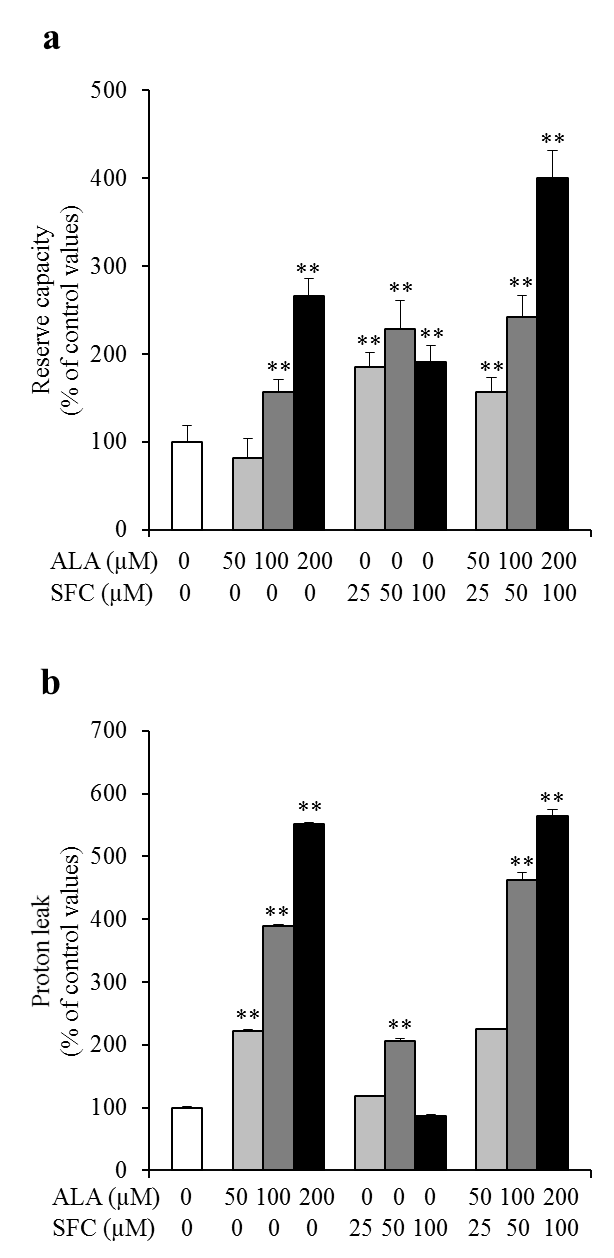


**Supplementary Figure 2**

Reserve capacity and proton leak of normal human skin fibroblasts treated with ALA or ALA/SFC increased in a concentration-dependent manner. Data are expressed as means ± SD of > 22 technical replicates, relative to the control. ** *P* < 0.01 vs. Control (Dunnett’s test)


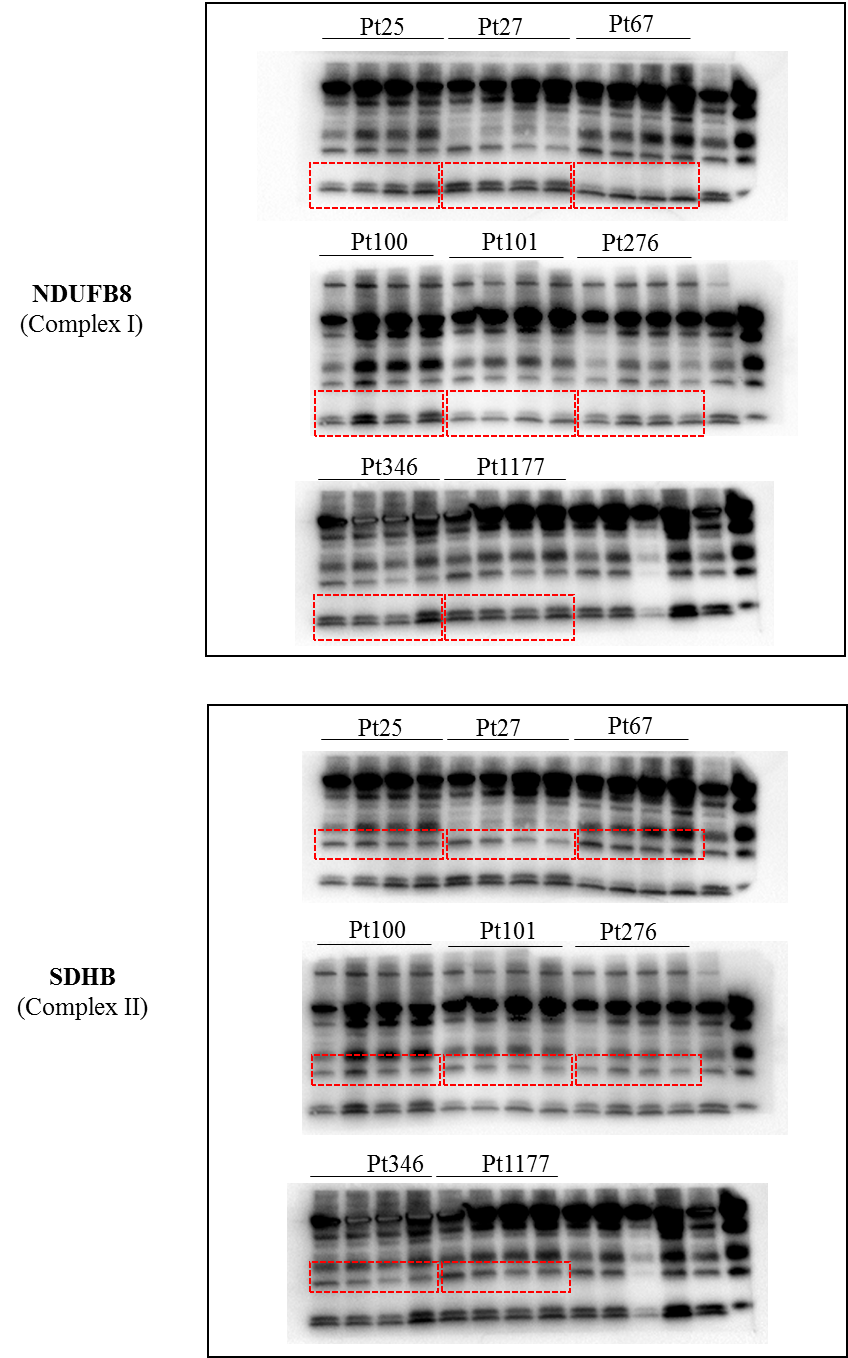


**Supplementary Figure 3. Full-length blots of Figure 4**

Red boxes indicate cropped regions shown in Figure 4.


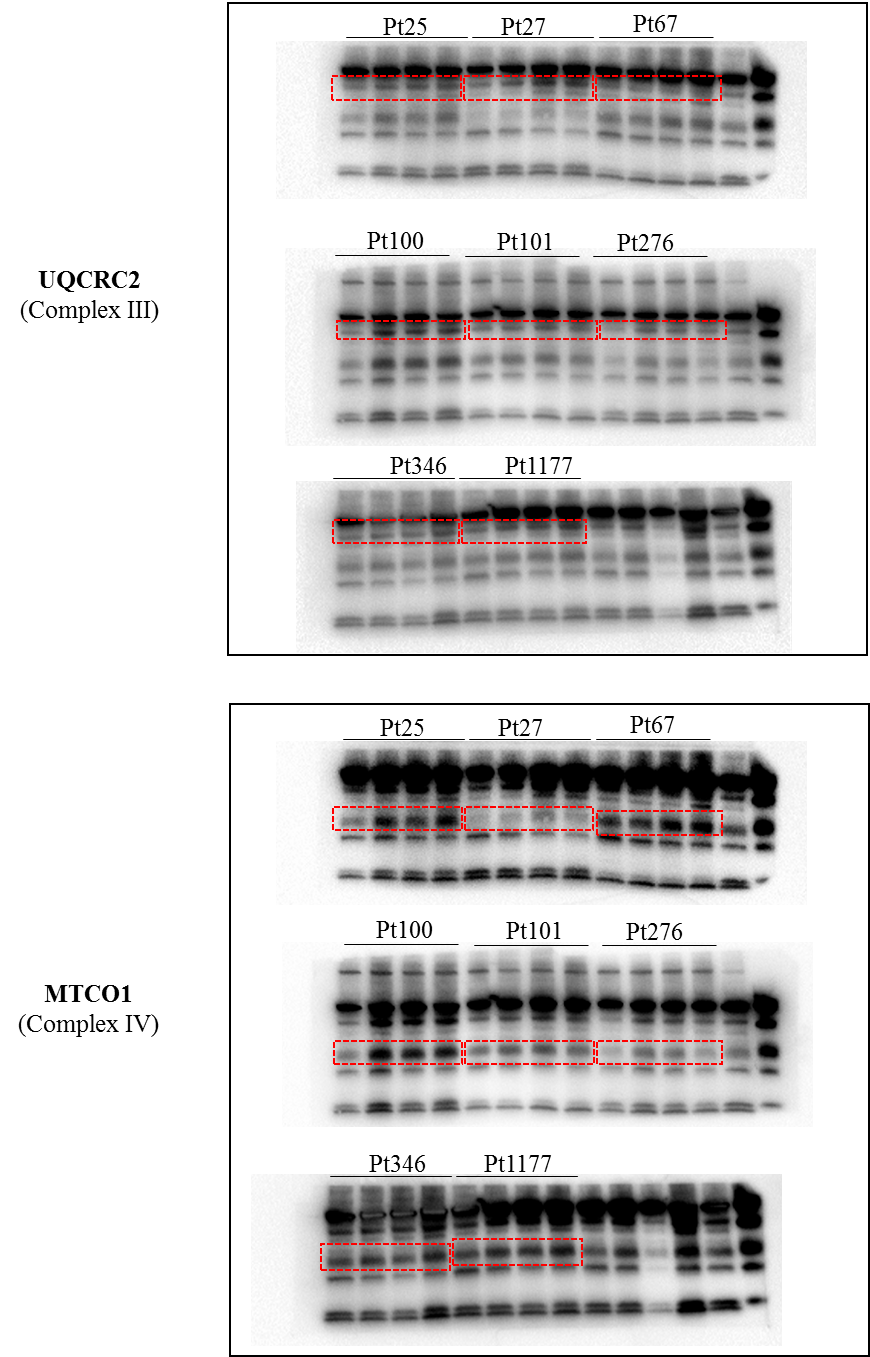


**Supplementary Figure 4. Full-length blots of Figure 4**

Red boxes indicate cropped regions shown in Figure 4.


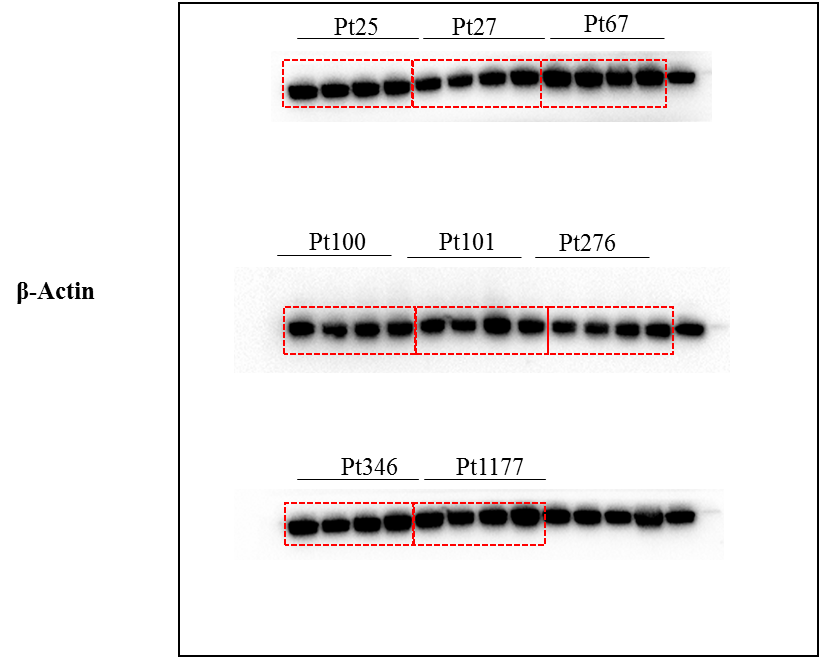


**Supplementary Figure 5. Full-length blots of Figure 4**

Red boxes indicate cropped regions shown in Figure 4.

**
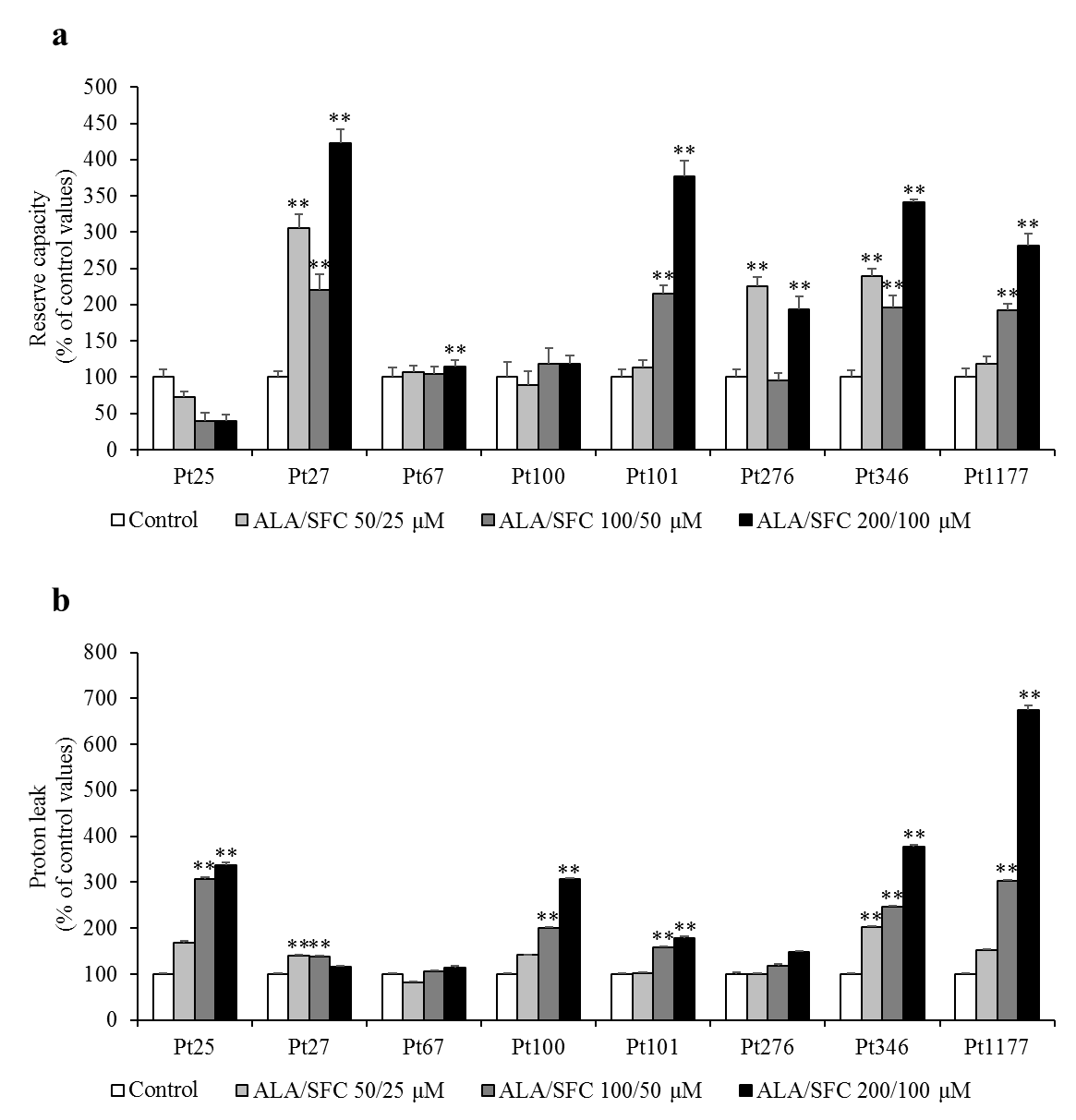
Supplementary Figure 6**

Reserve capacity and proton leak were significantly increased by ALA/SFC in 6 of 8 patient-derived fibroblasts. Data are expressed as means ± SD of > 14 technical replicates, relative to the control. ** P < 0.01 vs. Control (Dunnett’s test).


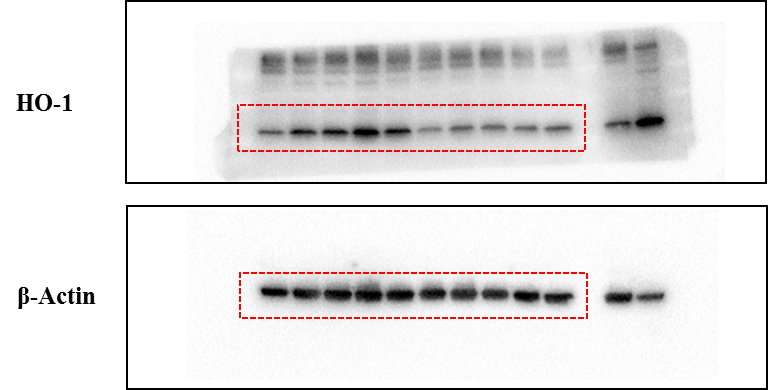


**Supplementary Figure 7. Full-length blots of Figure 6a**

Red boxes indicate cropped regions shown in Figure 6a.


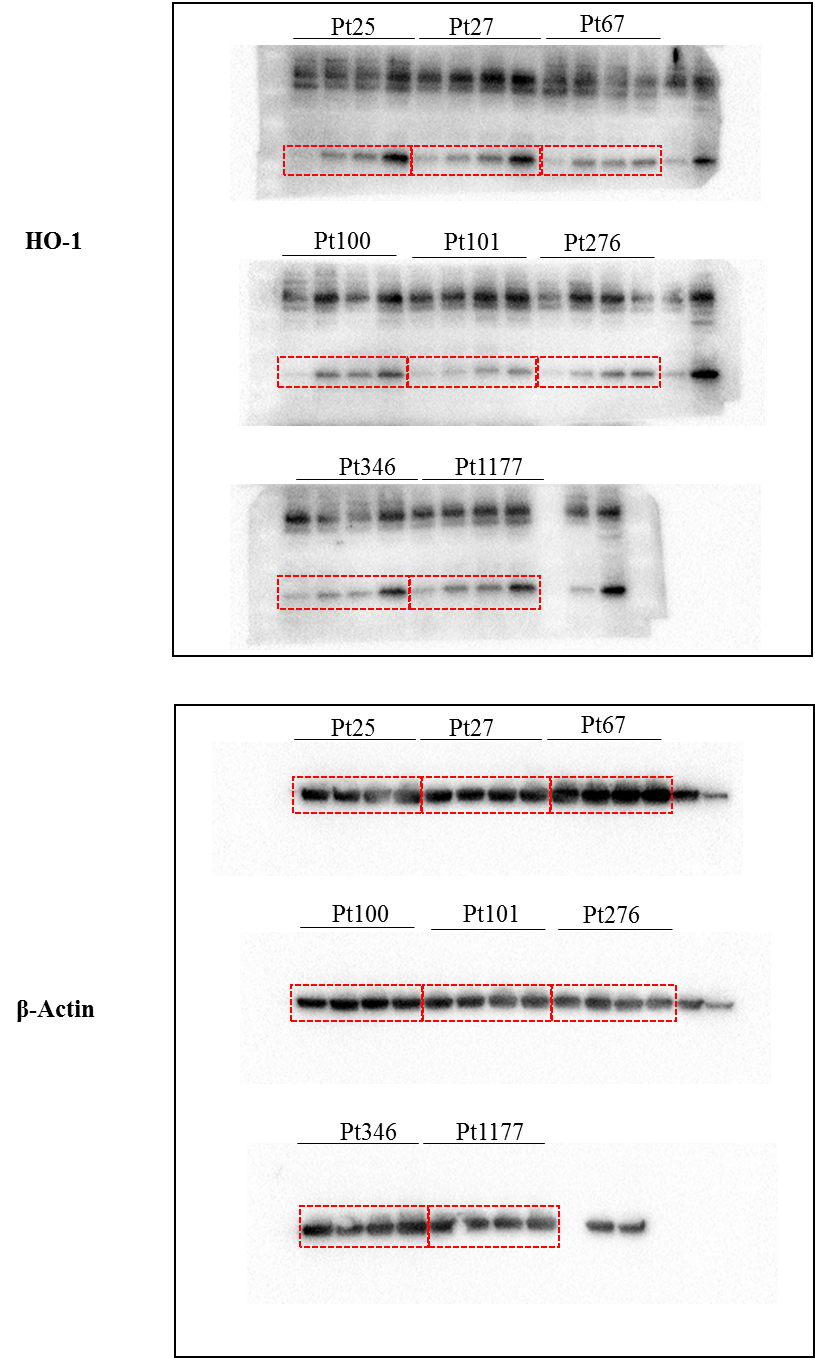


**Supplementary Figure 8. Full-length blots of Figure 6c**

Red boxes indicate cropped regions shown in Figure 6c.
